# The protective effect of DNase I in retinal vein occlusion

**DOI:** 10.17305/bb.2023.9780

**Published:** 2024-04-01

**Authors:** Guohua Deng, Xi Zou, Zhinan Liu, Hang Ren, Yanting Li, Bin Chen, Jun Zhang

**Affiliations:** 1 Department of Ophthalmology, Changzhou Third People’s Hospital, Changzhou Medical Center, Nanjing Medical University, Changzhou, China; 2 Department of Ophthalmology, The First Affiliated Hospital of Soochow University, Suzhou, China; 3 Department of Ophthalmology, Suzhou Eye and ENT Hospital, Suzhou, China

**Keywords:** Retinal vein occlusion (RVO), neutrophil extracellular traps (NETs), deoxyribonuclease I (DNase I)

## Abstract

Retinal vein occlusion (RVO) ranks as the second most prevalent retinal vascular disease, following diabetic retinopathy. Neutrophil extracellular traps (NETs) play an important role in vascular diseases. This study aimed to elucidate the relationship between NETs and RVO, and to discern the potential role of deoxyribonuclease I (DNase I) in the prevention and treatment of RVO through the modulation of NETs. We analyzed circulating NETs biomarkers, namely, cell-free DNA (cf-DNA), myeloperoxidase (MPO)-DNA, and neutrophil elastase (NE), in 30 RVO patients and 30 healthy individuals. We established an RVO mouse model using a retinal laser, and the mice were categorized into two groups: the DNase I group and the control group. Retinal images were taken at predetermined time points, and the state of the retinal vessels was assessed. Both tissue and blood samples were harvested for analysis of NETs expression through methods, such as western blotting, immunofluorescence staining, and enzyme-linked immunosorbent assay (ELISA). Our findings indicate an increase in circulating NETs biomarkers in human and mouse RVO cases, while also verifying the presence of NETs in the retinal thrombus of the RVO model. Both in vitro and in vivo tests revealed that DNase I attenuated NETs formation. Moreover, DNase I injections led to diminished NETs biomarker levels and a reduced duration of the thrombus after the RVO model establishment. Consequently, DNase I, a well-established modulator of NETs formation, might exhibit protective properties in the prevention and treatment of RVO.

## Introduction

Retinal vein occlusion (RVO) ranks as the second most common retinal vascular disease, following diabetic retinopathy [1]. Although the development of RVO is widely considered to be associated with vascular thrombosis, oxidative stress, and inflammation, the pathogenesis of RVO still requires further research. Patients diagnosed with RVO may develop vision loss or even blindness when complications, such as neovascular glaucoma and macular edema arise [2]. Currently, RVO complications, including non-perfused areas and neovascular abnormalities, are clinically treated with intravitreal injections of anti-vascular endothelial growth factors, anti-inflammatory glucocorticoids, and retinal laser photocoagulation [3, 4]. Retinal revascularization has undergone extensive research in RVO patients since the injection of a tissue plasminogen activator into the obstructed retinal vein was first reported [5]. However, the complexity of this surgical procedure is hindering its wide applicability in clinical settings.

Neutrophil extracellular traps (NETs) are net-like, extracellular bactericidal structures released by NETosis in response to pathogenic stimuli. They are composed of unwound DNA strands, histones, and bacteriostatic proteins [6]. NETs play a pivotal role in thrombosis pathogenesis, where their net-like structures provide a foundation for red blood cell and platelet aggregation, while their components activate the coagulation pathway [7]. Moreover, in cases of deep vein thrombosis (DVT), inhibiting NETosis has been demonstrated to confer protection [8]. A recent study observed increased NETs-related markers in the peripheral blood of RVO patients, suggesting a potential association between NETs and the development of RVO [9].

Deoxyribonuclease I (DNase I), an economical, convenient, and widely used NETs inhibitor, has rarely been used in the prevention and treatment of RVO. This study aimed to verify the presence of NETs in RVO and assess the protective efficiency of DNase I through in vitro and in vivo experiments, thereby providing a theoretical basis for RVO prevention and treatment.

## Materials and methods

### Patient selection and parameter testing

We conducted a case-control pilot study, including 30 RVO patients and 30 healthy individuals, between March 2022 and February 2023. The RVO diagnoses were made in accordance with the RVO Preferred Practice Pattern [10]. Following blood sample collection, patients were administered appropriate treatments. Participants were excluded from the study if they met any of the following criteria: (1) a history of thrombotic disorders or systemic inflammation; (2) a history of uveitis or iris rubeosis; (3) previous RVO treatment history; (4) hematological or connective tissue disorders, malignant tumors, or an inability to cooperate with the study’s requirements.

The coagulation function parameters, including activated partial thromboplastin time (APTT), fibrinogen (FIB), thrombin time (TT), and prothrombin time (PT), were assessed using an automatic coagulation function analyzer (SYSMEX, CA-1500), and the serum levels of myeloperoxidase (MPO) and neutrophil elastase (NE) were measured using commercially available enzyme-linked immunosorbent assay (ELISA) kits (MULTI SCIENCES, China; Abcam, USA). The cell-free DNA (cf-DNA) was measured using a method similar to ELISA. In brief, a standard calf thymus solution (Sigma-Aldrich, Germany) was prepared in a concentration gradient. A volume of 100 µL each of serum and calf thymus solution was added to 96-well plates. After a reaction period of 30 min with 100 µL of SYTOX green (ThermoFisher Scientific, USA), the fluorescence intensity was measured at 485 nm, from which the concentration of cf-DNA was calculated.

### In vitro experiment

Neutrophils were extracted from healthy volunteers using a neutrophil isolation kit (Beijing Solarbio Science & Technology, China). The cells were seeded onto a 12-well plate at a density of 1.5 × 10^6^ cells/well and were then equally divided into three groups. As described in previous studies, the DNase I group was treated with 100 U/mL DNase I (Beijing Solarbio Science & Technology, China) and 20 nM phorbol-12-myristate-13-acetate (PMA) [11]. The PMA group was treated solely with 20 nM PMA, while the remaining wells received Roswell Park Memorial Institute 1640 (RPMI 1640) medium stimulation. After a 4 h incubation, all groups underwent SYTOX green staining for 30 min. Subsequently, slides were photographed under an inverted fluorescence microscope (Leica DMILLED, Leica, Germany) and the cf-DNA levels in the culture medium were measured as previously described.

### Animal experiments

C57 mice were procured from JOINN Laboratories (Suzhou, China). Twenty eight-week-old male mice were randomly divided into RVO and control groups, with each group consisting of ten mice. NETs-related biomarkers were analyzed to confirm the differences between RVO and healthy groups. The RVO model was established as described below. Mice in the control group were exposed to an equal-energy laser between retinal vessels to account for any non-specific laser-induced damage. Following the establishment of the RVO model, serum MPO, NE, and cf-DNA levels were measured in both groups. In a separate setup, 74 eight-week-old male mice were randomly divided into DNase I and control groups, with each group consisting of 37 mice. Twelve mice were selected at random from each group for retinal imaging, while the remaining mice were set aside for sample collection at the different subsequent time points, as described below. The DNase I dosage was based on previous research. To elaborate, the DNase I group was administered an intravenous injection of 50 U/mouse DNase I both 1 h before and 24 h after the RVO model establishment. In contrast, the control group was injected with an equivalent volume of phosphate-buffered saline (PBS) [12].

### Retinal vein occlusion model establishment

After pupil dilation, the mice were administered an injection of 37.5 mg/kg Rose Bengal (Sigma-Aldrich, Germany) via the tail vein. Following anesthesia, the two thick retinal veins were irradiated with a 532 nm fundus laser (VISULAS 532 s, Carl Zeiss, Germany). The used laser parameters were: 100 mW laser power, 50 µm spot size, a distance of 375 µm from the spot to the optic disc, 100 µm spot spacing, 1000 ms single spot time, 1000 ms exposure duration, and three laser spots per vein.

### Image acquisition

Fundus images were acquired prior to laser therapy and at subsequent intervals of 4 h, 1 d, 3 d, and 8 d after establishing the RVO model. Three independent investigators analyzed the images to assess vessel obstruction. In instances of differing opinions, a vessel was deemed obstructed if at least two of the three investigators considered it so, otherwise, it was recorded as unobstructed. The images were acquired using the anterior segment imaging system, with a coverslip coated with ophthalmic gel placed in front of the eye under examination.

### Sample collection and storage

A certain volume of blood was drawn from the heart 0.5 h before and 4 h, 1 d, 3 d, and 8 d after the RVO model establishment. At each of these time points, five mice were randomly selected from each group for blood collection. For the serum samples, portions of the blood (500 µL) were transferred to 1.5 mL centrifuge tubes without anticoagulants. These samples were then allowed to rest for 60 min before being centrifuged at 3000 rpm for 10 min at 4 ^∘^C. Subsequently, the supernatant was collected and stored in centrifuge tubes at −80 ^∘^C.

In a preliminary experiment, NETs biomarkers peaked 24 h after laser therapy. As a result, eye samples were collected for histological evaluation at this time, along with the retinal protein extraction conducted at this time for western blotting analysis.

### Neutrophil extracellular traps biomarker monitoring

The concentrations of MPO-DNA complexes and NE in mice’s peripheral blood were assessed using an ELISA kit (MEIMIAN, China; Abcam, USA). Serum cf-DNA levels were determined after reacting with SYTOX green (ThermoFisher Scientific, USA) by comparing its absorbance to that of standard calf thymus (Sigma, USA). Additionally, a western blot of retinal proteins was conducted to analyze the citrullinated histone H3 (H3cit) and MPO (Abcam, USA).

### Ethical statement

All study procedures adhered to the Association for Research in Vision and Ophthalmology Statement for the Use of Animals in Ophthalmic and Vision Research. Furthermore, all study operations were conducted in accordance with the World Medical Association Declaration of Helsinki. The study protocol received approval from the Ethics Committee of Changzhou Third People’s Hospital (02A-A20210014). The clinical experiment was registered in the Chinese Clinical Trial Registry (ChiCTR2200056618). Written informed consent was obtained from all participants involved in this study.

### Statistical analysis

Images from immunofluorescence were analyzed using ImageJ. Statistical analyses were performed using Origin 2021 and SPSS 24.0. Continuous data were presented as mean ± standard deviation (SD). The differences between the two groups were analyzed using a non-paired *t*-test and a chi-squared test. A *P* value of < 0.05 was considered statistically significant.

## Results

### Participants’ characteristics

A total of 30 RVO patients and 30 healthy controls were included in this research. There were no significant differences in age, sex, presence of hypertension, diabetes, smoking, and drinking habits between the groups (*P* > 0.05). The indices of thrombotic function were increased in the RVO group (*P* < 0.05). The clinical characteristics and laboratory indices for both groups are presented in Table 1.

**Table 1:** Clinical characteristics and laboratory data of RVO cases and healthy controls

	**Control group**	**RVO group**	***P* value**
Sex (male)	50%	52%	0.796
Age (years)	61.6 ± 4.5	60.7 ± 3.9	0.466
Diabetes	10 (33.0%)	13 (43.3%)	0.426
Hypertension	14 (47.0%)	12 (40.0%)	0.602
Smoking	7 (23.3%)	13 (43.3%)	0.100
Drinking	6 (20.0%)	11 (36.6%)	0.152
PT (s)	12.4 ± 1.0	10.9 ± 1.4	< 0.001
APTT (s)	33.2 ± 3.2	31.4 ± 1.9	0.013
TT (s)	15.6 ± 1.4	15.5 ± 1.3	0.726
FIB (g/L)	3.1 ± 0.4	4.1 ± 0.9	< 0.001

Data are presented as *n* (%) or mean ± standard deviation. Comparison between groups was calculated using the unpaired *t*-test and chi-square exact test. RVO: Retinal vein occlusion; PT: Prothrombin time; APTT: Activated partial thromboplastin time; TT: Thrombin time; FIB: Fibrinogen.

### Increased neutrophil extracellular traps biomarkers in retinal vein occlusion

**Figure 1.**
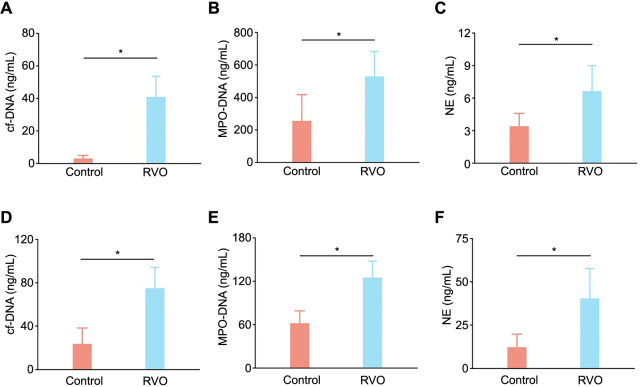
**Comparative levels of NETs biomarkers in RVO cases vs healthy controls.** (A–C) Levels of NETs biomarkers measured in human RVO cases and healthy controls. Circulating NETs biomarkers (cf-DNA [A], MPO-DNA [B], and NE [C]) were higher in RVO patients compared to the controls (*n* ═ 30 per group; *P < 0.05). (D–F) Levels of NETs biomarkers measured in mice RVO model group and healthy controls group. Circulating NETs biomarkers (cf-DNA [D], MPO-DNA [E], and NE [F]) were higher in the RVO model group compared to the control group (*n* ═ 10 per group; *P < 0.05). NETs: Neutrophil extracellular traps; RVO: Retinal vein occlusion; cf-DNA: Cell-free DNA; MPO: Myeloperoxidase; NE: Neutrophil elastase.

To ascertain the relationship between NETs biomarkers and RVO incidences, circulating NETs remnants, including cf-DNA, MPO-DNA, and NE concentrations were measured in both humans and the RVO mouse model. In comparison to the control group (healthy people), levels of cf-DNA, MPO-DNA, and NE were increased to 40.8 ± 12.8 ng/mL, 528.4 ± 154.3 ng/mL, and 6.6 ± 2.4 ng/mL, respectively (Figure 1A–1C). In the RVO model, circulating NETs remnants of cf-DNA, MPO-DNA, and NE were 74.8 ± 19.3 ng/mL, 124.8 ± 23.1 ng/mL, and 40.3 ± 17.3 ng/mL, respectively, showing a significant increase compared to the control group (Figure 1D–1F).

**Figure 2.**
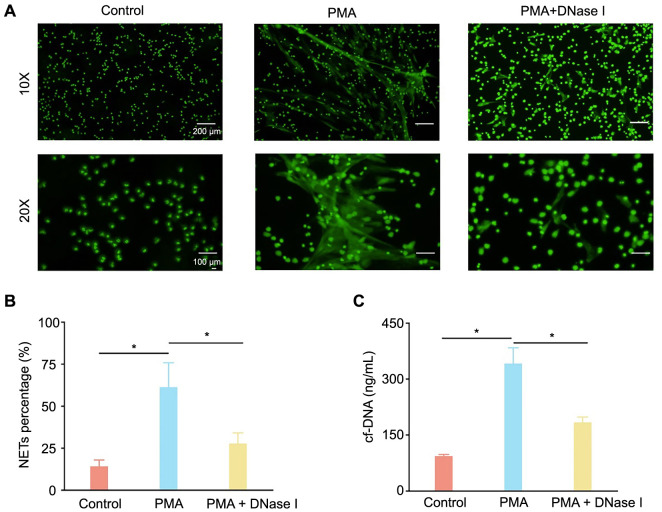
**DNase I attenuates NETs formation in vitro**. In the control group, the neutrophils were treated with RPMI 1640 for 4 h. In the PMA group, the neutrophils were stimulated with 20 nM PMA for a duration of 4 h. For the PMA + DNase I group, the neutrophils were treated with DNase I (100 U/mL) for 0.5 h before being exposed to PMA. (A) Immunofluorescence images of neutrophils under different conditions: control, PMA-stimulated, and PMA + DNase I-treated groups, with both 10× and 20× magnifications; (B) Quantitative analysis showcasing the percentage of NETs formation across the different groups (*n* ═ 5; *P < 0.05); (C) Bar graph illustrating the levels of cf-DNA in the culture medium across the different groups (*n* ═ 3; *P < 0.05). DNase I: Deoxyribonuclease I; RPMI 1640: Roswell Park Memorial Institute 1640 medium; PMA: Phorbol-12-myristate-13-acetate; NETs: Neutrophil extracellular traps; cf-DNA: Cell-free DNA.

### Effect of DNase I on neutrophil extracellular traps in in vitro experiments

Neutrophils were extracted, stimulated, and stained as previously described. Cells were stimulated with 20-nM PMA to induce NETs formation and DNase I (100 U/mL) was introduced to attenuate this induction (Figure 2A). Immunofluorescence images were captured at different magnifications. Five images at 10× magnification were randomly selected from each group for analysis using ImageJ. Significant differences in the area of NETs fluorescent staining were observed among the control, PMA, and DNase I groups (Figure 2B). The culture medium cf-DNA levels of the control, PMA, and PMA + DNase I groups were 93 ± 4.7 ng/mL, 341.4 ± 42.5 ng/mL, and 183.5 ± 14.6 ng/mL, respectively (Figure 2C).

### DNase I inhibits neutrophil extracellular traps formation in retinal vein occlusion mouse model

**Figure 3.**
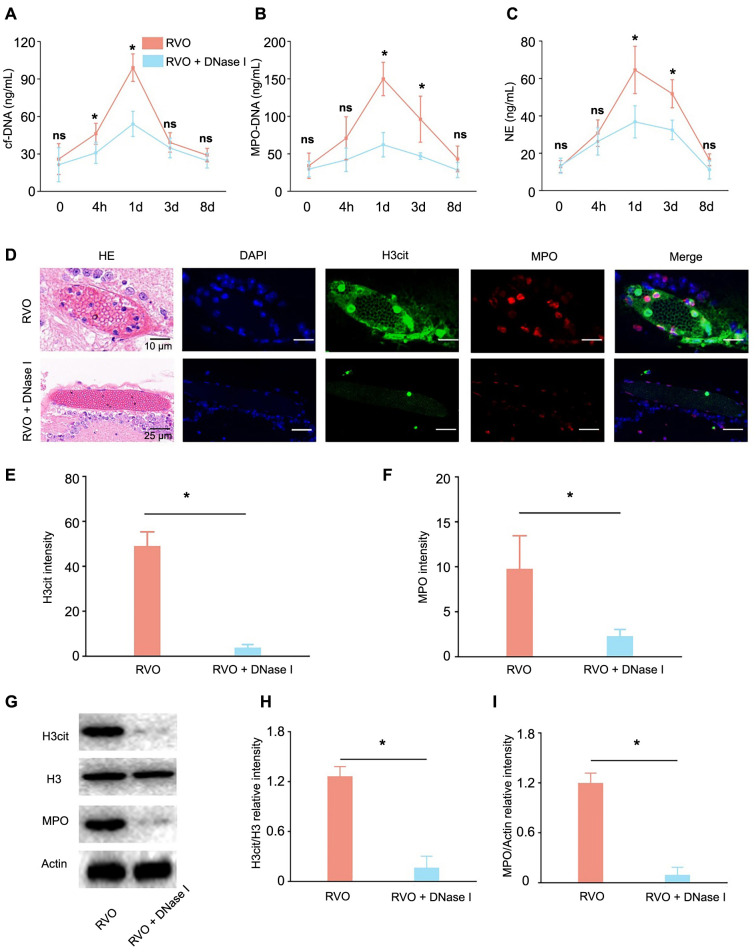
**DNase I attenuates NETs formation in vivo**. (A–C) Time-course graphs depicting the changes in circulating NETs biomarkers, cf-DNA(A), MPO-DNA (B), and NE (C), following retinal laser therapy, with the RVO + DNase I group showing diminished levels in comparison to the RVO group (*n* ═ 3; *P < 0.05). (D) Histological and immunohistological images showing decreased MPO and H3cit expression in RVO thrombus after the DNase I injection. (E and F) Semi-quantitative analysis of H3cit (E) and MPO (F) immunofluorescence intensity in both the RVO and RVO + DNase I groups (*n* ═ 3; *P < 0.05). (G) Western blot analysis of the H3cit and MPO relative protein expression in the retinas of both the RVO and RVO + DNase I groups. (H and I) Semi-quantitative analysis of the H3cit (H) and MPO (I) protein expression in both the RVO and RVO + DNase I groups (*n* ═ 3; *P < 0.05). DNase I: Deoxyribonuclease I; NETs: Neutrophil extracellular traps; cf-DNA: Cell-free DNA; MPO: Myeloperoxidase; NE: Neutrophil elastase; RVO: Retinal vein occlusion; H3cit: Citrullinated histone H3; ns: Non-significant; HE: Hematoxylin and eosin; DAPI: 4’,6-diamidino-2-phenylindole; H3: Histone 3.

After the retinal laser therapy, the cf-DNA, MPO-DNA, and NE concentrations increased in the mice’s peripheral blood. The NETs biomarkers in the RVO + DNase I group were notably lower than those in the RVO group (Figure 3A–3C). The peak levels of circulating NETs biomarkers were observed on the first day following the model establishment, and consequently, retinal proteins and eyeballs were collected for western blot analysis and fluorescent immunostaining. The expression of NETs-related markers, including MPO and H3cit, within the retinal thrombus, were lower after the DNase I injection (Figure 3D–3F). Western blot analysis revealed an increase in MPO and H3cit levels after the retinal laser procedure, which was counteracted by DNase I, suppressing this increase (Figure 3G–3I).

### DNase I shortens the duration of the thrombus existence in retinal vein occlusion

**Figure 4.**
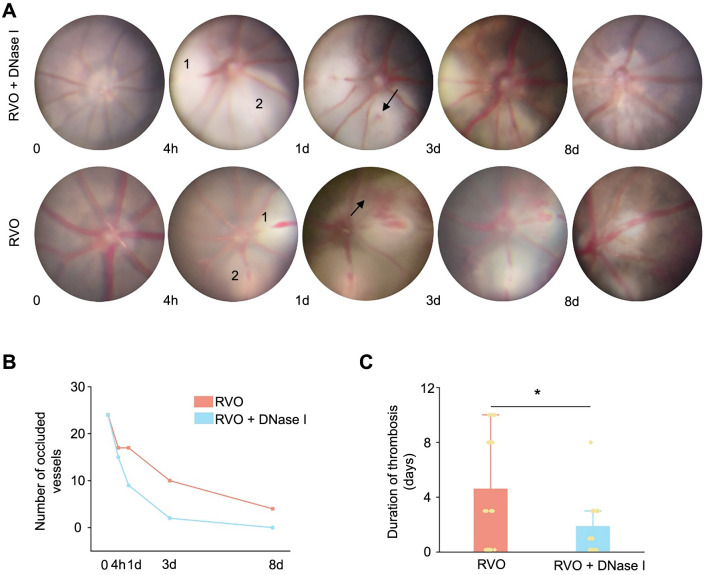
**DNase I shortens the duration of the RVO thrombus**. (A) Representative retinal pictures of RVO and RVO + DNase I groups taken at different time intervals post-laser treatment, with “0” indicating before laser treatment. Veins labeled as “1” and “2” are the ones that received laser irradiation, and retinal bleeding can be observed (indicated by an arrowhead); (B) Graph depicting the number of occluded retinal vessels over time for both groups; (C) Duration of thrombus presence in retinal vessels for both RVO and RVO + DNase I groups (*n* ═ 24; *P < 0.05). DNase I: Deoxyribonuclease I; RVO: Retinal vein occlusion.

In mice subjected to retinal laser therapy, fundus images were acquired before the treatment and at intervals of 4 h, 1 d, 3 d, and 8 d after the treatment. In the RVO model, observations included vessels that were not occluded, completely occluded vessels, patchy bleeding, and reperfusion subsequent to occlusion (Figure 4A). The state of the occlusion changed within the first 10 min after retinal laser therapy and remained stable during the first 24 h [13]. Consistent with previous research, most occlusions experienced reperfusion by the eighth day after irradiation [14], and our findings indicate that DNase I accelerated the reperfusion (Figure 4B). Moreover, the average duration of the retinal thrombus in the RVO + DNase I group was 1.85 ± 2.23 days, which was less than that observed in the RVO group (Figure 4C).

## Discussion

As a common retinal vascular disease, RVO is prone to causing visual impairment and even blindness due to complications. While many studies have focused on the treatment and complications of RVO, and factors like inflammation and oxidative stress are believed to be associated with RVO, the detailed mechanisms are not fully understood [15]. NETs are net-like DNA structures released by neutrophils through the NETosis mechanism, containing MPO, H3cit, NE, and DNA as their components [9, 16]. Moreover, MPO-DNA, cf-DNA, and NE, widely utilized as NETs-related markers [17, 18], were selected to assess the NETs levels in the peripheral blood. Meanwhile, MPO and H3cit were analyzed using western blot and immunofluorescence techniques. Previous studies have shown that NETs can stimulate intrinsic and extrinsic coagulation pathways [19], leading to platelet activation, adhesion, and aggregation, while the interaction between NETs and platelets can lead to a vicious cycle of NETs formation and platelet activation [20]. Additionally, NETs can recruit red blood cells, promote fibrin deposition, induce thrombosis, and enhance the stability of the venous thrombus [19]. Recently, elevated levels of NETs-related markers were found in the peripheral blood of RVO patients [9], a finding that is consistent with our clinical experiment results. In addition, NETs might promote numerous coagulant factors and thrombosis [21, 22], therefore, we evaluated the thrombotic function indices and found that the average FIB level in RVO patients exceeded the normal range, suggesting a potential association with the NETs status.

The exact role of NETs in RVO still necessitates powerful and direct evidence, including analyses of retinal tissues. Pondering whether inhibiting NETs might shorten the duration of retinal thrombus and alleviate RVO symptoms, our team developed an RVO mouse model for further research. 

During our animal experiments, after the establishment of the RVO model, circulating NETs levels increased on day 1, with a subsequent decrease over the following days. This pattern might have been related to the thrombus stability within the first 24 h [13], and the continuous production of NETs. As the days passed and the occluded vessels began to recanalize, circulating NETs levels decreased. A German study detected the presence of NETs in DVT, and demonstrated that DNase-I could curb DVT progression by limiting NETs [8]. In our research, DNase I effectively decreased NETs expression both in the retina and peripheral blood. Notably, the difference in NETs reduction appeared more pronounced in the retina, possibly due to its inherently low baseline expression of NETs-related biomarkers. Furthermore, utilizing DNase I resulted in a reduced duration of retinal thrombosis compared to the control group. These findings indicate that NETs could play an important role in RVO and that DNase I might emerge as a novel preventive and therapeutic option for RVO. Nonetheless, although NETs are known to initiate and propagate venous thrombosis and promote fibrous vascular occlusion in chronic thrombosis [8, 23], their specific role in RVO demands further in-depth research. Interestingly, our data revealed that the number of smokers in the RVO group was nearly double that of the control group, offering another intriguing avenue for future research.

This study had some limitations that future research should address. Firstly, while the finding indicates an association between the NETs and RVO development and suggests a beneficial role for DNase I in the treatment of RVO, the relationship between different DNase I doses and in vivo NETs levels needs exploration to determine the most effective dose for RVO prevention or treatment. Secondly, due to the unavailability of certain tools, some RVO-related assessments, such as optical coherence tomography (OCT), were not conducted in the RVO model. Incorporating larger sample sizes and utilizing DNase I knocked out (KO) mice could enhance the robustness of the evidence. Lastly, a deeper understanding of the exact pathways and mechanisms through which DNase I treats RVO is still necessary.

## Conclusion

In conclusion, the plasma NETs remnants were found to be significantly increased in RVO patients. In our animal experiments, upon successful establishment of the RVO model, NETs biomarkers showed a significant increase. With the injection of DNase I, NETs formation was inhibited, making the thrombus more unstable. This indicates a potential new target for RVO treatment. Further studies and clinical trials are necessary to identify the exact mechanism by which DNase I, in inhibiting NETs, may aid in the prevention and treatment of RVO.

## Data Availability

Datasets analyzed during this study are available upon reasonable request from the corresponding author, Jun Zhang.
